# Using structural equation modeling to examine correlations between health-related physical fitness and cell health among Chinese college students

**DOI:** 10.1186/s12889-024-19067-8

**Published:** 2024-06-10

**Authors:** Jian Zhu, Yi Yang, Yanmin Zhao, Guoyang Qin, Xin Su

**Affiliations:** 1 Physical Education Department, Nanjing University of Aeronautics and Astronautics, Nanjing, China; 2 College of Physical Education, Hengxing University, Qingdao, China; 3 Department of Physical Education, China University of Petroleum (East China), Qingdao, China; 4 College of Physical Education, Shandong Normal University, Jinan, China; 5 Department of Physical Education, Shenzhen Polytechnic, Shenzhen, China

**Keywords:** Structural equation model, Physical fitness, Cell health, College students, Health

## Abstract

**Introduction:**

College students’ physical fitness is likely to be directly related to their cells’ health. However, there is a lack of literature on whether the relationship between cell health and college students’ physical fitness is direct or indirect. This study used a structural equation modeling (SEM) approach to investigate the connection between cell health and college students’ physical fitness.

**Methods:**

This cross-sectional study collected data from 838 volunteers (502 males and 336 females, average age of 18.74 ± 1.5 years) who were college students from the Shandong province of China in July 2023. Initially, we obtained anthropometric measurements and conducted physical fitness tests on the students. Then, we performed Pearson correlation analysis and principal component analysis to screen variables and explore potentially influencing factors. Finally, we examined associations between the variables and determined whether there were direct or indirect influences among factors using SEM.

**Results:**

The results revealed a significant correlation between the cell health factor and the muscle strength factor (path coefficient = 0.97; *p* < 0.001) as well as the fat obesity factor (path coefficient = -0.52; *p* < 0.001). The cardiovascular factor exhibited a weak correlation with the cell health factor (path coefficient = 0.11; *p* < 0.01). Moreover, the cardiovascular factor acted as a mediating variable between the muscle strength factor and the cell health factor, with a positive correlation observed between the muscle strength factor and the cell health factor (path coefficient = 0.40; *p* < 0.001).

**Conclusion:**

These findings suggest that cell health is indicative of muscle strength and cardiorespiratory fitness. Our findings demonstrate that assessing the cell health of college students can be a valuable method for evaluating their overall health.

## Background

Most college students undergo a crucial transitional period from adolescence to adulthood, characterized by significant developments in lifestyle and behavior. This transitional phase presents a unique opportunity to study and influence long-term health-related physical fitness (HPF) outcomes [1]. Consequently, the Health-related Physical Fitness (HPF) level of college students is likely to significantly impact their future lifestyle and overall health. While recent research has increasingly focused on the health-related physical fitness (HPF) of college students [2], it primarily examines the relationship between physical activity and HPF. There is a noticeable gap in research concerning innovative methods to measure HPF, particularly methods that integrate cellular health indicators such as phase angle (PhA). In addition to physical activity, HPF is influenced by various other factors, such as body weight, spirometry, and the standing long jump. Due to its usefulness as an indicator of overall health, the development of effective and reliable methods of evaluating the HPF of college students is an important research issue.

Different procedures and evaluation criteria for measuring college students’ HPF have been established in different parts of the world. In China, the HPF is primarily measured using the Chinese National Student Physical Fitness Standard (CNSPFS), which incorporates measurements of physical form (height and weight), physical function (spirometry), and physical fitness (sit and reach flexibility test, standing long jump, chin-ups or sit-ups, and the 50 m and 800 m/1000m run) [3]. In other countries, such as the United States of America, the HPF is measured using the American College of Sports Medicine (ACSM) health-related physical fitness test, which assesses cardiovascular fitness, muscular strength and endurance, flexibility, and body composition. While the CNSPFS utilizes fewer indicators compared to the ACSM health-related physical fitness test, the components of both tests are similar [4]. However, neither test includes cell health indicators such as the extracellular fluid rate or phase angle (PhA) [5]. Derived from bioelectrical impedance analysis, PhA is an emerging indicator that provides deep insights into cell health and integrity. It has been effectively used to assess the nutritional status and muscle mass quality in athletes, where a higher PhA is associated with better cell membrane integrity and correlates positively with greater muscle strength and quality [6]. Furthermore, PhA’s relevance extends beyond muscle metrics to include metabolic functions such as insulin resistance and blood glucose levels [7, 8]. Considering its proven effectiveness in these areas, PhA has the potential to serve as a non-invasive marker in college students, monitoring not only nutritional status but also predicting their capacity for higher physical performance and improved health outcomes. Integrating PhA into health-related physical fitness (HPF) assessments could significantly enhance their predictive power by adding a cellular health dimension, thus offering a more comprehensive understanding of overall health metrics in the college population. This study hypothesizes that PhA could similarly serve as a vital health indicator in assessing the physical fitness of college students, filling a critical gap in current HPF assessments. Therefore, this study aims to explore the utility of incorporating cell health indicators, specifically PhA, into the assessment frameworks of college students’ HPF. We propose a comprehensive framework that integrates PhA with traditional HPF measures to provide a holistic view of student.

Although the inclusion of cell health indicators in the assessment of the physical fitness of college students may prove invaluable, their relationship with other tests remains unclear, particularly whether they have direct and indirect correlations with other tests. Hence, this study aims to propose and examine a framework for assessing cell health and investigating its relationship with other indicators. This framework may provide insights into the association between cell health, physical fitness, and other indicators.

## Conceptual framework

In exploring the HPF of college students, we propose a structural equation model (SEM) to systematically assess the influencing factors and their interactions. This model includes five main latent variables: Muscle Strength, Obesity, Cardiovascular Health, Endurance Running, and Cell Health. Each latent variable is measured by specific indicators, such as grip strength and standing long jump for Muscle Strength, and BMI, relative fat value, etc., for Obesity. Within the model, Cell Health is posited as a core regulatory variable, hypothesized to directly influence the other four health dimensions. For instance, PhA, an indicator of cell health, is presumed to predict muscle quality and strength and is also associated with fat percentage and cardiovascular health status. Additionally, Cardiovascular Health is assumed to directly affect endurance running performance, while Obesity could indirectly impact endurance performance through its effect on cardiovascular function. This conceptual model not only allows us to evaluate the relationships between various health indicators but also reveals how these relationships collectively influence the overall health and physical fitness of college students. By employing this multidimensional approach, we can gain a more comprehensive understanding of the complex factors affecting college student health and provide a scientific basis for designing more effective health promotion strategies.

## Materials and methods

### Participants

The population for the present study was Chinese male and female firstyear college students enrolled in year from 2022 to 2023. A convenience sampling strategy was used for recruiting universities in Shandong Province of China to provide archival data for this study. In the end, only one university agreed to participate [China University of Petroleum (East China)]. This cross-sectional study collected physical test data from 1,123 volunteers (after inviting 1,500 potential participants, a total of 1,123 volunteers agreed to participate in this study, 702 males and 421 females, resulting in an actual participation rate of 74.9%) were enrolled in variety of departments (e.g., finance, engineering and science) and physical education, which is a compulsory class for every student in the university. Inclusion criteria: college students aged 17 to 20 years old. Exclusion criteria: (1) Individuals with long-term health issues or physical disabilities that affect their ability to perform physical fitness tests. (2) who have had major surgery or a serious injury in the past six months. (3) who are unable to provide written informed consent. (4) who have safety concerns about participating in any of the physical fitness tests or are advised against participating by a doctor. The sample size is based on the rule of thumb. All the participants provided written informed consent. For participants under the age of 18 years, both their informed consent and that of their parents or guardians were obtained. The study protocol was approved by the Shandong Normal University Institutional Research Commission (Approval number: 2,023,054, may, 2023, Jinan, Shandong, China), and all procedures were conducted in accordance with the Declaration of Helsinki.

### Procedure

By following the technical specifications, including those for conducting CNSPFS evaluations. We conducted tests for various physical fitness indexes and, finally performed the cardiorespiratory endurance test. While the other tests were only conducted once, we utilized data from the best of three jumps for the standing long jump test and the fastest of two runs for the 50-m running test. All data collection was supervised by a specially trained research team to ensure the accuracy and consistency of the data. A two-day workshop was conducted prior to the commencement of the study, where data collectors were instructed on the proper techniques for administering physical fitness tests, handling equipment, and ensuring participant compliance. The training included practical sessions to familiarize the team with the assessment protocols and to address any procedural queries. To manage the data collection efficiently among the all volunteers, the measurement work was organized into several sessions spread over a month. Each session was scheduled to handle approximately 40 students per day to maintain a manageable workload and ensure precise data collection. Volunteers were grouped based on their availability and informed in advance about their scheduled times. Each measurement session lasted around three hours, during which each volunteer underwent a series of assessments as per the study protocol.

### Test program

#### Body composition analysis

The body composition data in this study were obtained using a high-quality body composition analyzer (SECA, UK, mBCA515). Indicators that provide information on health status were selected, including phase angle, phase angle percentile, relative fat mass, fat-free mass, visceral fat mass, skeletal muscle mass, total energy expenditure, resting energy expenditure, body water percentage, extracellular water percentage, hip circumference, and waist circumference. Of particular interest is the phase angle, which serves as a direct testing variable reflecting the structure, function, nutritional reserves, and severity of diseases in human cells [5]. A high phase angle value indicates excellent nutritional status, intact cell membrane function, and a high body cell count, while a lower value suggests poor nutritional status, significant cell membrane damage, and impaired cell function. The normal range of phase angle values for males and females is 5.1 to 7.9 and 4.5 to 7.3, respectively [9].

#### BMI calculation

Volunteers provided their own height and weight data. The body mass index (BMI) was calculated using the following formula: BMI = weight (kg) / height (m^2^). Students were categorized into four groups based on their BMI values according to the criteria recommended by the World Health Organization (WHO) as follows: <18.5 kg/m^2^, 18.5 ~ 23.9 kg/m^2^, 24 ~ 27.9 kg/m^2^, and ≥ 28 kg/m^2^, representing underweight, average weight, overweight, and obese individuals, respectively [10].

### Physical fitness test

#### 50 m sprint

The objective of the 50-m sprint test was to evaluate students’ speed and the power of their lower limbs. Students were tested in groups of four. When the investigator indicated “go”, the subjects began the 50 m sprint [9]. They were asked to complete the entire race as quickly as possible, and their times were recorded in minutes and seconds. A Fusion Smart Speed System was used as the timing device (Fusion Sport, Coopers Plains, QLD, Australia). The CNSPFS recommends that men aged 17–20 should be able to perform 9.1 s, and women in the same age range should aim for 10.3 s [10].

#### Sit and reach flexibility test

To assess lower back flexibility, the sit and reach test was conducted. Each subject sat on the test instrument and was barefoot. They gradually reached forward as far as possible with their knees extended. The test was conducted twice, with the best of the two scores being retained [10]. The CNSPFS recommends that men aged 17–20 should be able to perform 3.7 cm, and women in the same age range should aim for 6.0 cm [10].

#### Standing long jump

The standing long jump was conducted to assess lower-limb power. Each participant stood at the starting line and was asked to jump forward as far as they could. The distance of the jump was measured in meters from the starting line to the heel of the foot closest to the line. The test was conducted twice, and the best score out of the two attempts was retained [11]. The CNSPFS recommends that men aged 17–20 should be able to perform 208 cm, and women in the same age range should aim for 151 cm [10].

#### 800–1000 m run

Each student stood at the starting line and was asked to complete the 800-m or 1000-m run as fast as possible. The time for each run was measured and recorded in minutes and seconds. Female students ran the 800-m distance, while male students ran the 1000-m distance [11]. The CNSPFS recommends that men aged 17–20 should be able to perform 272 s, and women in the same age range should aim for 274 s [10].

#### Pull-ups

Pull-ups were used to evaluate the participants’ upper body muscular strength. The test was scored based on the number of pull-ups each individual performed. The subjects were required to execute standard pull-ups. This test was only performed by male students [11]. The CNSPFS recommends that men aged 17–20 should be able to perform 10 pull-ups [10].

#### Bent-leg sit-ups

Each subject was directed to lie on a mat with their knees bent at a 90-degree angle and then to raise their upper body and touch their knees with their elbows. The number of bent-leg sit-ups completed in 1 min was recorded. This test was only performed by female students [11]. The CNSPFS recommends that women aged 17–20 should be able to perform 26 repetions [10].

#### Cardiovascular test

Systolic blood pressure, diastolic blood pressure, and heart rate were measured in accordance with medical testing standards. The tests were conducted twice at the same time of day for three consecutive days, and the average of the measurements was used as each participant’s overall test score.

#### Vital capacity weight index

The vital capacity, measured using the XF495-KDL model apparatus (Beijing Baiwan Electronic Technological Apparatus Center, China), was utilized to evaluate participants’ physiological capacity. Students had to stand before the apparatus, hold the handle properly, and position their mouths on the blowpipe. They then pressed the button, took a deep breath, and exhaled completely. The apparatus automatically calculated the maximal breathing capacity. The vital capacity weight index was calculated by dividing the measured vital capacity by a participant’s weight. The CNSPFS recommends that men aged 17–20 should be able to perform 3100 ml, and women in the same age range should aim for 2000 ml [10].

#### Maximum grip strength

The maximum grip strength of each participant’s dominant hand was measured with an accuracy of 0.1 kg using a hydraulic manual dynamometer (GRIP-D TKK 5401, Takei Scientific Instruments CO, Tokyo, Japan). The CNSPFS recommends that men aged 17–20 should be able to perform 35 kg, and women in the same age range should aim for 20 kg [10].

### Statistical analysis

Experimental data were processed using the SPSS (version 26.0, Chicago, IL, USA). All data were presented in the “mean ± standard deviation” (M ± SD) format. The Kolmogorov-Smirnov test, along with histograms and Q-Q plots were used to assess the normal distribution of the data.For normally distributed data, pearson correlation analysis was used to calculate the correlation coefficient of the measured variables and to identify those with the strongest associations. For variables that were not normally distributed, we considered using Spearman rank correlation analysis. After standardizing the relevant variables, principal component analysis and the direct oblimin method (direct oblique method) were utilized for exploratory factor analysis. A strong correlation between the variables and the principal components is indicated when the absolute value of the factor loadings of each indicator exceeds 0.5. Finally, structural equation modeling (SEM) was employed to explore both the direct and indirect associations among the variables. To assess the goodness-of-fit of SEM, we used several commonly accepted fit indices, including the Goodness of Fit Index (GFI), Standardized Root Mean Square Residual (SRMR), Root Mean Square Error of Approximation (RMSEA), Parsimonious Normed Fit Index (PNFI), Parsimonious Goodness of Fit Index (PGFI), Normed Fit Index (NFI), Non-Normed Fit Index (NNFI) and Comparative Fit Index (CFI). Specifically, we adopted the following reference values as indicators of a good fit: GFI, NFI, NNFI and CFI values greater than 0.90, PNFI and PGFI values greater than 0.50, SRMR and RMSEA values less than 0.08 [11]. Statistical significance was set at a p-value of < 0.05.

## Results

Of the initial sample of 1,123 volunteers, 838 first-year students (502 males and 336 females) with a mean age of 18.74 ± 1.5 years completed all the tests, including the physical fitness tests. Table 1 presents the descriptive statistics of the data, showcasing the characteristics of the variables. It is worth noting that all the variables exhibited a normal distribution (Kolmogorov-Smirnov test, *p* > 0.05).

**Table 1:** Descriptive statistics of constituent factors

Variable	Men	Women	Total Mean ± SD
Phase angle	5.20 ± 0.52	5.40 ± 0.64	5.32 ± 0.57
Phase angle percentile	10.10 ± 15.41	14.50 ± 18.84	12.30 ± 16.87
BMI (kg/m^2^)	22.06 ± 2.76	20.11 ± 3.37	21.78 ± 3.02
Relative fat value (%)	18.31 ± 7.32	14.79 ± 8.94	16.82 ± 8.01
Non-fat content (kg)	59.04 ± 7.76	49.51 ± 9.48	54.43 ± 8.49
Visceral fat content (L)	1.24 ± 0.54	1.22 ± 0.66	1.24 ± 0.59
Skeletal muscle content (kg)	28.22 ± 4.50	22.34 ± 5.50	25.49 ± 4.92
Total energy expenditure (kcal/kg/day)	2951.29 ± 312.57	2329.11 ± 382.06	2640.17 ± 342.13
Resting energy expenditure (kcal/kg/day)	1855.17 ± 186.80	1426.99 ± 228.33	1641.08 ± 204.47
Body water rate	0.72 ± 0.05	0.49 ± 0.07	0.60 ± 0.06
Extracellular fluid rate	43.67 ± 1.74	38.36 ± 2.12	41.05 ± 1.90
Vital capacity index (ml/kg^2^)	0.93 ± 0.24	0.82 ± 0.29	0.89 ± 0.26
Standing long jump (m)	218.97 ± 26.28	157.55 ± 32.12	203.52 ± 28.76
Sit and reach (cm)	7.10 ± 7.32	9.50 ± 8.95	8.55 ± 8.013
Grip strength (kg)	40.40 ± 8.19	25.40 ± 10.01	36.44 ± 8.96
800–1000 m performance (s)	271 ± 40.18	240 ± 49.11	255.65 ± 43.98
50 m performance (s)	7.90 ± 0.19	8.50 ± 0.23	8.4 ± 0.21
The mean of two systolic blood pressures (mmHg)	119.26 ± 10.21	117.13 ± 12.48	118.62 ± 11.18
The mean of two diastolic blood pressures (mmHg)	68.04 ± 6.58	68.11 ± 8.04	68.15 ± 7.20
The mean of two quiet heart rates (beats/min)	79.62 ± 12.20	83.49 ± 14.91	82.44 ± 13.35
Waist circumference (m)	0.77 ± 0.07	0.75 ± 0.09	0.76 ± 0.08

### Correlation analysis

Pearson correlation analysis was conducted on the selected variables, revealing that among the 21 variables, there were weak correlations between diastolic blood pressure and ten other variables including phase angle (*r* = 0.18), phase angle percentage (*r* = 0.11), non-fat content (*r* = 0.20), skeletal muscle content (*r* = 0.13), total energy consumption (*r* = 0.06), resting energy consumption (*r* = 0.07), extracellular fluid rate (*r* = 0.14), standing long jump (*r* = 0.14), grip strength (*r* = 0.03),, and the 50 m run (*r* = 0.20); the sit and reach test with BMI (*r* = 0.07), relative fat rate (*r* = 0.04), non-fat content (*r* = 0.16), skeletal muscle content (*r* = 0.18), resting energy consumption (*r* = 0.05), body water percentage(*r* = 0.06), and extracellular fluid rate (*r* = 0.02) for seven variables; and the 1000 m/800m run with non-fat content (*r* = 0.14) and skeletal muscle content (*r* = 0.16) for two variables. Besides these, significant correlations existed among all other variables. Particularly strong correlations were found between non-fat content, total energy consumption (*r* = 0.99), resting energy consumption (*r* = 0.90), and skeletal muscle content (*r* = 0.91), and similarly, waist circumference and BMI index also showed very strong correlations (*r* = 0.82); due to the similarity of the measures they represent, the former can be considered indicators reflecting skeletal muscle content, and the latter as common indicators measuring obesity. Therefore, in the next step of exploratory factor analysis, skeletal muscle content and BMI index are used to replace the aforementioned similar indicators.

### Exploratory analysis of constituent factors

Exploratory factor analysis was conducted using principal component analysis and the direct oblimin method. The results showed that the Kaiser–Meyer–Olkin (KMO) coefficient was 0.769, the chi-square value of the sphericity test was 15435.481, df was 153, and *p* < 0.001, indicating that the data were suitable for a factor analysis. After performing oblique rotation using eigenvalues greater than one as the basis for forming factors, a corresponding factor loading matrix was obtained (as shown in Table 2). Accoding the factor loadings of each indicator (> 0.5), five common factors were extracted: (i) muscle strength factor, (ii) fat and obesity factor, (iii) cardiovascular factor, (iv) endurance running factor and (v) cell health. These factors collectively explained 83.93% of the total variation. The components of each factor are shown in Table 3.

**Table 2:** The factor loadings of each indicator

Variable	Factors				
	Muscle strength	Obesity	Cardiovascular	Cell health	Endurance running
Phase angle	0.31	0.01	-0.01	0.81	-0.02
Phase angle percentile	-0.25	0.16	-0.04	0.93	0.17
BMI	0.30	0.88	-0.02	0.13	0.01
Relative fat value	-0.44	0.82	0.04	-0.06	0.04
Visceral fat content	0.12	0.72	0.05	0.13	-0.27
Skeletal muscle content	0.86	0.27	-0.03	0.15	-0.05
Body water rate	0.44	-0.76	-0.06	0.03	-0.01
Extracellular fluid rate	-0.34	0.32	-0.03	-0.66	0.20
Vital capacity index	-0.20	-0.82	0.03	0.03	0.01
Standing long jump	0.67	-0.39	0.01	0.20	0.02
Sit and reach	-0.07	0.05	0.16	0.14	0.75
Grip strength	0.75	0.01	0.06	0.03	0.14
800–1000 m performance	-0.19	0.19	0.18	0.06	-0.63
50 m performance	-0.73	0.33	0.01	-0.18	0.02
Systolic blood pressures	0.48	0.20	0.67	0.01	0.07
Diastolic blood pressures	0.08	0.04	0.89	-0.02	0.07
Quiet heart rates	-0.30	-0.19	0.68	-0.03	-0.10
Eigenvalues	5.83	3.96	1.74	1.27	1.05
Explained Variance (%)	32.38	22.00	13.66	10.07	5.82
Cumulative Explained Variance (%)	35.38	54.38	68.04	78.11	83.93
Reliability	0.95	0.90	0.79	0.80	0.70

**Table 3:** Constituent factors on college students’ physical health

Factor	Element	Correlation
Muscle strength	Skeletal muscle content	0.86
	Standing long jump	0.67
	Grip strength	0.75
	50 m run	-0.73
Obesity	BMI	0.88
	Relative fat value	0.82
	Visceral fat content	0.72
	Body water rate	-0.76
	Vital capacity index	-0.82
Cardiovascular	Systolic blood pressures	0.67
	Diastolic blood pressures	0.89
	Quiet heart rates	0.68
Endurance running	Sit and reach	0.75
	800–1000 m	-0.63
Cell health	Phase angle	0.81
	Phase angle percentile	0.93
	Extracellular fluid rate	-0.66

### Model construction, fitness test, and validation

After comparing the mixed models, we observed that one mixed model containing the 800–1000 m run and the sitting trunk flexion variables, one mixed model featuring the 800–1000 m run variable, and another mixed model with only the sitting trunk flexion variable produced the following chi-square values relating to the mixed models: 1552.17, 480.345, and 1001.771, respectively. The mixed model comprising the 800-m or 1000-m run as an observation variable had the lowest chi-square value among the models, indicating best fitness.

**Fig. 1:**
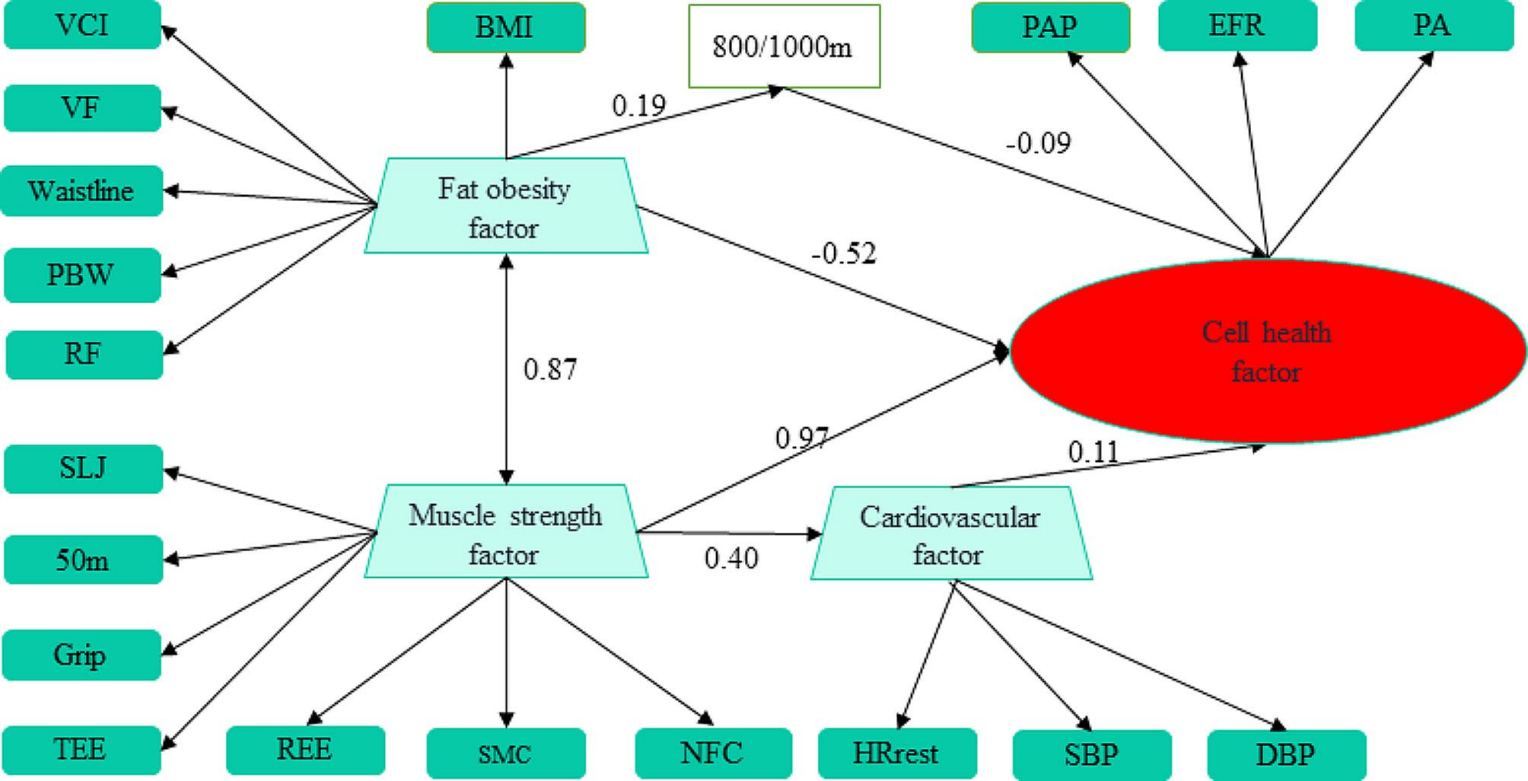
The SEM of health-related physical fitness and cell health. Note: VCI, vital capacity index; VF, visceral fat; BWR, body water rate; RF, relative fat; SLJ, standing long jump; TEE, total energy expenditure; REE, resting energy expenditure, SMC, skeletal muscle content; NFC, non-fat content; SBP, systolic blood pressures; DBP, diastolic blood pressures; PAP, phase angle percentile; EFR, extracellular fluid rate; PA, phase angle

Figure 1 showcases the detailed relationships among observed, latent, exogenous, and endogenous variables. The endogenous variable of cell health factor is primarily associated with the muscle strength factor, with a path coefficient of 0.97 (*p* < 0.001), and it is adversely impacted by the fat obesity factor, which has a path coefficient of -0.52 (*p* < 0.001). The cardiovascular function factor is weakly correlated with the cell health factor, with a path coefficient of 0.11 (*p* < 0.01). Furthermore, it serves as a mediating variable between the muscle strength and cell health factors, is positively correlated with muscle strength, and holds a path coefficient of 0.40 (*p* < 0.001).

The goodness of fit was assessed using the following statistical values: the value of χ ^2^/df, goodness-of-fit index (GFI), standardized root mean square residual (SRMR), root mean square Error of approximation (RMSEA), parsimony normed fit index (PNFI), parsimony goodness-of-fit index (PGFI), normed fit index (NFI), non-normed fit index (NNFI), comparative fit index (CFI). As illustrated in Table 4, all the indicators reached ideal levels, and the overall fitness between the model and data was excellent.

**Table 4:** Fitting index of the mixed model of the relationship between muscle robustness, fat obesity, cardiovascular function, endurance, and cell health

	Absolute fit				Reduced fit			Value-added fit		
	χ^2^/df	GFI	SRMR	RMSEA		PNFI	PGFI	NFI	NNFI	CFI
Evaluation criterion	< 3	> 0.9	< 0.08	< 0.08		> 0.5	> 0.5	> 0.9	> 0.9	> 0.9
Result	2.813	0.977	0.0514	0.0596		0.729	0.602	0.924	0.945	0.961

Note: goodness-of-fit index (GFI), standardized root mean square residual (SRMR), root mean square Error of approximation (RMSEA), parsimony normed fit index (PNFI), parsimony goodness-of-fit index (PGFI), normed fit index (NFI), non-normed fit index (NNFI), comparative fit index (CFI)

## Discussion

This study found there is a direct and positive correlation between cell health indicators, such as muscle strength and cardiovascular factors, and overall health. Additionally, it was found a negative correlation between cell health and fat obesity factor. The results of the study indicate that assessing cell health is a valuable method for evaluating the overall health of university students.

The structural equation model analysis revealed a significant positive correlation between cellular health, muscle strength, and cardiovascular factors, suggesting that cellular health is associated with the strength and endurance abilities of college students. Muscle strength is an important aspect of the physical health of college students and has become a crucial determinant of individuals’ overall physical health, with skeletal muscle mass being the most critical factor [12]. Previous studies suggested that muscle strength can independently predict overall mortality in humans [13]. Muscular fitness, including muscle strength and muscle mass, is crucial for overall human physical well-being. According to the ACSM, muscle strength and endurance are elements of muscle fitness, which is recognized as a component of health-related fitness in the context of promoting and maintaining the quantity and quality of physical activity [14]. Muscle strength is negatively correlated with the likelihood of experiencing injuries in daily life, physical activities, and sports, as well as with obesity, metabolic disease risk, cardiovascular disease risk, low back pain, and other factors [15–17].

In this study, the HPF level of college students was assessed using the cellular health factor, which demonstrated a strong correlation with scores in the CNSPFS for physical form (height and weight), physical function (spirometry), and physical fitness (sit and reach flexibility test, standing long jump, chin-ups or sit-ups, and the 50-m and 800-m or 1000-m run). Consequently, the findings are significant because they suggest that the HPF of college students can be utilized in health evaluations to facilitate interventions and preventive measures. Measures of cellular health, which are correlated with muscular strength and endurance measures can be employed to improve the physical health of college students. Through exploratory factor analysis, it was discovered cellular health factors are associated with adiposity measures such as BMI, pulmonary function index, visceral fat, and waist circumference. Obesity is a major contributing factor to low levels of physical health [18]. Correlation analysis revealed a negative correlation between the cell health indicator and the fat obesity factor, providing a framework for partially explaining the well-documented negative association between cell health and higher levels of body fat.

The evaluation of fat and obesity factors, including BMI, relative fat content, visceral fat, body water rate, and vital capacity index, is crucial for assessing overall health risks in college students. Historical data indicate that the average college student experiences a weight gain of approximately 1.55 kg over four years [19]. Such trends underscore the relevance of cell health indicators, which not only reflect physical fitness but also help establish the relationship between physical activity and overall health-related physical fitness (HPF). This study reinforces the importance of integrating comprehensive metrics such as BMI, pulmonary function index, and visceral fat levels into routine health assessments to provide a more holistic view of student health.

Comparisons of the mixed models revealed that the cell health factor, an endogenous variable, primarily correlates with the muscle strength factor. Furthermore, the study found that the cardiovascular function factor acts as a mediating variable between the muscle strength and the cell health factor. The results of the current study suggest that improving both muscle strength and aerobic capacity enhances cell health and physical fitness in college students, which aligns with the findings of previous studies. For example, Schmidt et al. found that short-duration high-intensity circuit training may improve muscle endurance, muscle strength, and aerobic fitness in moderately fit populations [20]. Previous studies have also indicated that excessive body mass can hinder performance due to additional load and restricted movement. Moreover, overweight or obese individuals may require higher levels of energy to engage in physical activities compared to those within the normal weight range. This increased energy demand can potentially discourage participation in such activities, though it is important to note that multiple factors contribute to physical activity levels and that the relationship between obesity and activity avoidance is complex and influenced by psychological, social, and physical variables [21].

This study has several limitations. The study sample might not accurately represent the characteristics of all university students in China due to the participants being from the same university. Conducting studies with larger sample sizes that include participants from various provinces and universities, as well as different cohorts with diverse area, lifestyle, age, and sex characteristics, could offer additional insights and help validate the results of this study. Finally, it is important to note that this study relied on cross-sectional data; thus, causal relationships between variables need to be investigated through longitudinal controlled trials.

## Conclusion

This study examined the relationship between cell health indicators and the fat and obesity, muscle strength, cardiovascular, and endurance factors. It discovered a direct and positive correlation between cell health indicators and other health indicators, such as the muscle strength and the cardiovascular factor. These findings indicate that cell health can serve as a valuable indicator of muscle strength and cardiorespiratory fitness. Additionally, the study identified a negative correlation between the cell health indicator and the fat obesity factor, highlighting the well-established association between cell health and body fat. The study results imply that the cell health indicator can be a helpful tool for assessing the health of university students.

## Data Availability

The data that support the findings of this study are not openly available due to reasons of sensitivity and are available from the corresponding author upon reasonable request.
